# Antibody-Mediated Therapies in Urothelial Carcinoma: A Critical Narrative Review

**DOI:** 10.3390/cancers18172743

**Published:** 2026-08-24

**Authors:** Se-Il Go, Viozzy Florendinha da Costa Xavier, Myoung Hee Kang, Won Sup Lee

**Affiliations:** 1 Department of Internal Medicine, Institute of Medical Science, Gyeongsang National University Changwon Hospital, Gyeongsang National University College of Medicine, Changwon 51472, Republic of Korea; gose1@gnu.ac.kr (S.-I.G.); maykang@gnu.ac.kr (M.H.K.); 2 Department of Internal Medicine, Institute of Medical Science, Gyeongsang National University Hospital, Gyeongsang National University College of Medicine, 15 Jinju-daero 816 Beon-gil, Jinju 52727, Republic of Korea; viozzyxavier@yahoo.com

**Keywords:** urothelial carcinoma, antibody–drug conjugates (ADCs), immune checkpoint inhibitors (ICIs), combination therapy, drug resistance, biomarkers

## Abstract

Urothelial carcinoma (UCC) remains a clinical challenge due to its high recurrence rate and aggressive advanced-stage behavior. Driven by a high tumor mutational burden and the consistent overexpression of surface antigens, including Nectin-4, Trop-2, and HER2, UCC has become a prime target for antibody-mediated therapies. Although both immune checkpoint inhibitors (ICIs) and antibody–drug conjugates (ADCs) show significant clinical efficacy, their rational combination further enhances antitumor activity via immunogenic cell death. However, therapeutic resistance and immune evasion still limit response durability in many patients. This review provides a comprehensive overview of the molecular principles, mechanisms of action, and clinical efficacy of antibody-based UCC therapies. Additionally, we analyze biomarkers for patient selection, the mechanisms underlying resistance development, and the rationale for ADC-ICI combinations. By evaluating current challenges and unmet needs, this review aims to clarify the evolving therapeutic landscape and suggest future directions for optimizing UCC treatment.

## Simple Summary

1. Treatment outcomes for urothelial carcinoma have significantly improved recently due to advancements in antibody-based therapies, particularly immune checkpoint inhibitors (ICIs) and antibody-drug conjugates (ADCs). 2. ICIs enhance the patient’s immune response against cancer cells, whereas ADCs selectively deliver cytotoxic drugs to tumor cells expressing specific surface proteins such as Nectin-4, Trop-2, and HER2. 3. However, not all patients respond, and treatment resistance and toxicity remain significant challenges. 4. This review summarizes the mechanisms, clinical efficacy, biomarkers, and resistance mechanisms of antibody-mediated therapies in urothelial carcinoma, and discusses novel ADCs, combination strategies, and personalized treatment approaches that can improve sustained clinical outcomes.

## 1. Introduction

Urothelial carcinoma (UCC), which accounts for approximately 90% of bladder cancers, has an age-standardized incidence of roughly 9.5 per 100,000 men and 2.4 per 100,000 women worldwide, with hundreds of thousands of new cases diagnosed annually [1,2]. Although not among the most common malignancies, UCC poses significant clinical challenges due to its high recurrence rate, aggressive behavior in advanced stages, and poor prognosis [3,4].

Biologically, UCC is a heterogeneous malignancy characterized by a complex molecular landscape with direct therapeutic implications. Genomic profiling studies have revealed a high tumor mutational burden (TMB), frequent chromosomal instability, and recurrent alterations in pathways related to cell cycle regulation, DNA damage response, and receptor signaling [5,6,7]. These features not only drive tumor aggressiveness and therapeutic resistance but also create vulnerabilities that can be exploited by antibody-mediated therapies. Immunologically, UCC exhibits a relatively high neoantigen load, which underlies the clinical activity of immune checkpoint inhibitors (ICIs) targeting the PD-1/PD-L1 axis by restoring antitumor T-cell function [8,9]. Nevertheless, immune evasion mechanisms, including impaired antigen presentation, adaptive immune resistance, and an immunosuppressive tumor microenvironment, limit response durability in a substantial proportion of patients [10,11]. These observations emphasize the need for antibody-based strategies that extend beyond checkpoint blockade and directly target tumor-intrinsic vulnerabilities.

Furthermore, UCCs are ideal targets for antibody–drug conjugates (ADCs) because they consistently and highly express tumor-associated cell surface antigens that are minimally expressed in normal tissues [12]. Nectin-4, Trop-2, and HER-2 are among the most extensively validated targets, with widespread expression across molecular subtypes. When antibodies bind to these antigens, selective intracellular uptake of cytotoxic payloads is possible, allowing for the combination of tumor specificity and potent anticancer activity [13,14]. The clinical success of ADCs targeting these molecules exemplifies how molecular profiling has translated directly into therapeutic innovation. Furthermore, mechanistic understandings of oncogenic signaling, DNA damage response, and immune regulation have informed rational combination strategies, such as pairing ADCs with ICIs to enhance antitumor efficacy via immunogenic cell death and increased antigen presentation [14,15]. Despite their encouraging efficacy, there are several clinically significant factors that can limit the therapeutic effect of antibody-mediated therapies in UCC [16]. Therefore, to maximize therapeutic efficacy, it is essential to clearly understand the types of agents across ICIs, ADCs, and combination therapies, as well as the mechanisms of action of each drug, functional combination therapies, and mechanisms of drug resistance.

In recent years, UCC has emerged as a paradigm tumor type for the development and clinical integration of antibody-mediated therapies, including ICIs, ADCs, and their combinations. These therapies are demonstrating meaningful clinical effects. However, limitations in therapeutic efficacy and resistance remain issues. Therefore, this review comprehensively examines antibody-mediated therapies for UCC, focusing on molecular principles, mechanisms of action, clinical efficacy, drug resistance, and potential solutions. By synthesizing these data, we aim to clarify the evolving role of antibody-based therapies and suggest future directions for their optimal application in the treatment of UCC.

## 2. Biological Rationale for Antibody-Mediated Therapy in Urothelial Carcinoma

### 2.1. Molecular Characteristics of UCC

UCC exhibits a high degree of genomic and immunologic heterogeneity, which has direct implications for antibody-based therapies. Key molecular features include:

- High tumor mutational burden (TMB): Frequent somatic mutations generate neoantigens that can be recognized by T cells, supporting the use of ICIs [17,18].
- Surface antigen overexpression: Tumor cells frequently express Nectin-4, Trop-2, and HER-2 at high levels, while normal tissue expression is limited, providing selective targets for ADCs [19,20,21].
- Recurrent pathway alterations: Changes in FGFR, PI3K/AKT, and cell-cycle genes contribute to tumor proliferation and survival, indirectly informing combination strategies [22].
- Immunological landscape: UCC displays immune-infiltrated (“hot”) and immune-desert (“cold”) phenotypes, affecting ICI responsiveness and guiding therapy selection [23,24].

### 2.2. Targetable Surface Antigens for ADC

An ideal antibody target exhibits high tumor specificity, cell-surface accessibility, internalization upon antibody binding, and sufficient expression across clinically relevant tumor populations. In UCC, Nectin-4, TROP-2, and HER2 represent the most clinically advanced ADC targets, whereas several additional surface antigens are being investigated as potential therapeutic targets [25,26,27,28,29,30].

- **Nectin-4:** Expressed across most molecular subtypes; target of enfortumab vedotin [25].
- **Trop-2:** Frequently overexpressed; target of sacituzumab govitecan [26,27].
- **HER2:** Subset of tumors; target of disitamab vedotin (RC48) [28,29,30].

The high prevalence and membrane expression of Nectin-4, Trop-2, and HER2 support the therapeutic rationale for ADCs that selectively deliver cytotoxic payloads while sparing normal tissue [19,20,21].

Emerging targets: Several additional surface antigens are being investigated to expand the therapeutic reach of ADCs beyond Nectin-4, TROP-2, and HER2. HER3 (ERBB3) is an emerging target because of its frequent expression and its role in receptor signaling and therapeutic resistance. HER3-directed ADCs such as patritumab deruxtecan and bispecific EGFR×HER3 ADCs such as izalontamab brengitecan are undergoing clinical evaluation, including studies enrolling patients with urothelial carcinoma [31,32].

- Cadherin-6 (CDH6) is another potential ADC target because of its tumor-associated expression and capacity for antibody-mediated internalization. Early clinical development of CDH6-targeting ADCs has provided proof of concept for this approach, although clinical development has primarily focused on other solid tumors rather than urothelial carcinoma [33].
- SLITRK6 was identified as a urothelial cancer-associated surface antigen and has been investigated using the ADC AGS-15E (sirtatumab vedotin), which delivers MMAE to SLITRK6-expressing tumor cells. A phase I study demonstrated clinical activity in heavily pretreated metastatic urothelial carcinoma, supporting SLITRK6 as a potential therapeutic target [34,35].
- B7-H3 (CD276) is another emerging tumor-associated antigen that is overexpressed in several solid malignancies, including urothelial carcinoma. B7-H3-directed ADCs, including ifinatamab deruxtecan, are being evaluated in early-phase clinical studies that include patients with bladder cancer [36,37].

Collectively, these emerging targets illustrate the expanding repertoire of antibody-mediated therapeutic strategies in urothelial carcinoma. However, unlike Nectin-4 and TROP-2, and despite promising early clinical or preclinical findings, HER3, CDH6, SLITRK6, and B7-H3 remain investigational targets in urothelial carcinoma, and their optimal biomarker thresholds, sequencing, and clinical positioning have yet to be established.

## 3. Categories of Antibody-Mediated Therapy in Urothelial Carcinoma

Antibody-mediated therapies in UCC can be broadly classified into immune checkpoint inhibitors (ICIs), antibody–drug conjugates (ADCs), and emerging antibody-based strategies, each with distinct mechanisms, clinical applications, and limitations.

### 3.1. Immune Checkpoint Inhibitors (ICIs)

ICIs restore T-cell-mediated antitumor immunity by blocking the PD-1/PD-L1 inhibitory axis, reversing T-cell exhaustion, and reinvigorating cytotoxic T-cell function in the tumor microenvironment [38]. PD-1 is expressed on activated T cells, while PD-L1 is frequently upregulated on urothelial carcinoma (UCC) cells and tumor-associated immune cells, representing an adaptive immune-evasion mechanism [39]. Engagement of PD-1 by PD-L1 suppresses T-cell proliferation, cytokine production, and effector function [40]; ICIs disrupt this interaction to restore antitumor immunity.

Approved ICIs in UCC include:

- Anti-PD-1: Pembrolizumab, Nivolumab;
- Anti-PD-L1: Atezolizumab, Durvalumab, Avelumab.

Pivotal trials such as KEYNOTE-045, CheckMate 275, and IMvigor210 demonstrated durable responses and improved overall survival in a subset of patients, particularly those with high PD-L1 expression [41,42,43]. Overall response rates remain modest (~15–25%), but responders can experience long-term benefit. However, primary or acquired resistance is common, and PD-L1 expression alone is an imperfect biomarker due to tumor heterogeneity and dynamic regulation [44]. This underscores the need for improved biomarker strategies and combination therapies.

### 3.2. Antibody–Drug Conjugates (ADCs)

Antibody–drug conjugates (ADCs) combine tumor-targeting monoclonal antibodies with potent cytotoxic payloads via specialized linkers, enabling selective delivery to cancer cells while limiting systemic exposure [45]. Among the leading ADCs in urothelial carcinoma (UCC), Enfortumab Vedotin (EV), Sacituzumab Govitecan (SG), and Disitamab Vedotin (RC48) illustrate key variations in target selection, cytotoxic payloads, and linker strategies (Table 1) [25,26,27,28,29,30].

Enfortumab vedotin (EV) is composed of a humanized IgG1 monoclonal antibody directed against Nectin-4, a cell adhesion molecule consistently overexpressed in urothelial carcinoma [25]. It employs a protease-cleavable linker to attach monomethyl auristatin E (MMAE), a microtubule-disrupting agent. This configuration allows selective internalization and release of MMAE within Nectin-4-positive tumor cells, resulting in cytotoxicity predominantly via mitotic arrest.

Sacituzumab govitecan (SG) targets Trop-2, a transmembrane glycoprotein broadly expressed across UCC subtypes [26]. In contrast to EV, SG is conjugated to SN-38, the active metabolite of irinotecan, via a hydrolyzable CL2A linker and is characterized by a relatively high drug-to-antibody ratio (DAR ~7–8). SN-38 exerts cytotoxicity through topoisomerase I inhibition, leading to DNA damage and apoptosis. SG demonstrates a strong bystander effect due to partial extracellular payload release [27].

Disitamab vedotin (DV) utilizes a humanized anti-HER2 antibody conjugated with MMAE via a protease-cleavable linker similar to EV [28]. Although HER2 expression is heterogeneous in UCC, a clinically relevant subset of patients—including those with HER2-low tumors—may derive benefit, likely due to the bystander effect of MMAE. Phase II studies have demonstrated promising activity in platinum-refractory HER2-positive UCC [29,30].

Next-generation Nectin-4-directed ADCs: The clinical success of enfortumab vedotin has stimulated the development of next-generation Nectin-4-directed ADCs. Bulumtatug fuvedotin (BFv; 9MW2821) is a next-generation Nectin-4-targeting ADC that delivers MMAE and has demonstrated encouraging antitumor activity in patients with advanced solid tumors, including urothelial carcinoma, in a first-in-human phase I/II study [46]. In subsequent studies of advanced urothelial carcinoma, BFv demonstrated substantial antitumor activity as monotherapy and has also shown promising efficacy in combination with the PD-1 inhibitor toripalimab [47,48]. In an updated phase Ib/II analysis of BFv plus toripalimab, the ORR was 83.0% and confirmed ORR was 74.5% among 47 evaluable patients, while the treatment-naïve subgroup had an ORR of 87.5% and confirmed ORR of 80.0% [48]. These findings support further clinical development of next-generation Nectin-4-directed ADC–ICI combinations. However, because these studies were not randomized comparisons with EV and differed in patient populations and treatment settings, the apparent numerical differences in response rates should not be interpreted as evidence of superiority over EV.

Beyond Nectin-4, TROP-2, and HER2, several emerging ADC targets are under investigation in urothelial carcinoma. The HER3-directed ADC patritumab deruxtecan is being evaluated in the phase II HERTHENA-PanTumor01 study, which includes a bladder cancer cohort, whereas the EGFR×HER3 bispecific ADC izalontamab brengitecan has demonstrated preliminary activity in a phase II study of advanced urothelial carcinoma [31,32]. The SLITRK6-targeted ADC AGS-15E has demonstrated early clinical activity in metastatic urothelial carcinoma [35]. B7-H3-directed ADCs such as ifinatamab deruxtecan are also undergoing early-phase clinical development in advanced solid tumors, including bladder cancer [37]. Other targets, such as CDH6, remain at an earlier stage of development and have primarily been investigated in ovarian, renal, and other non-urothelial solid tumors [33,49].

## 4. Clinical Impact of ADC Therapies

The clinical activity of major antibody–drug conjugates (ADCs) and their combinations with immune checkpoint inhibitors (ICIs) in urothelial carcinoma (UCC) is summarized in Table 2. Single-agent ADCs such as enfortumab vedotin (EV), sacituzumab govitecan (SG), and disitamab vedotin (DV) provide meaningful activity in later-line, treatment-refractory settings:

- EV achieves objective response rates (ORR) of ~40–50%, with median progression-free survival (PFS) of 5.8–6.5 months and median overall survival (OS) of 11.7–12.8 months [50,51]. Importantly, EV demonstrated a significant improvement in OS and PFS compared with chemotherapy, supporting its established role in previously treated advanced UCC [51].
- SG demonstrated ORRs of 27–32% and median OS of 12–13 months, primarily in later-line therapy [52,53]. However, these findings were not confirmed in the randomized phase III TROPiCS-04 trial. Although SG produced a higher ORR than physician’s choice chemotherapy (23% vs. 14%), it did not significantly improve OS (10.3 vs. 9.0 months; HR, 0.86; 95% CI, 0.73–1.02; *p* = 0.087) or PFS (4.2 vs. 3.6 months) [54]. Following these findings, the FDA withdrew the urothelial carcinoma indication for SG on 22 November 2024 [55]. Thus, the clinical role of SG in previously treated UCC should be interpreted cautiously in light of the negative phase III findings [54].
- DV demonstrates ORRs of 50–55% with median OS around 14 months in HER2-expressing UCC [29]. However, unlike EV, the efficacy of DV has not yet been established in a large randomized phase III trial. Accordingly, its promising activity should be interpreted in the context of the predominantly single-arm evidence base and the dependence of treatment benefit on HER2 expression.

### Antibody-Mediated Therapies in the Peri-Operative Setting

The clinical development of antibody-mediated therapies in urothelial carcinoma (UCC) is increasingly moving from metastatic disease toward earlier, potentially curative settings. In muscle-invasive bladder cancer (MIBC), peri-operative treatment aims to eradicate micrometastatic disease, improve pathological response, and reduce the risk of recurrence following radical cystectomy. This paradigm has been supported by accumulating evidence for both immune checkpoint inhibitors (ICIs) and ADC–ICI combinations.

Early-phase studies established the feasibility and antitumor activity of neoadjuvant ICI therapy. The phase II PURE-01 trial demonstrated a pathological complete response (pCR) rate of 42% following neoadjuvant pembrolizumab before radical cystectomy, with long-term follow-up subsequently reporting 5-year event-free and overall survival rates of 68% and 77%, respectively [56,57]. These findings provided an important rationale for further evaluation of antibody-based immunotherapy in the peri-operative setting.

Adjuvant ICI therapy has also demonstrated clinical benefit in high-risk MIBC. In the CheckMate 274 trial, adjuvant nivolumab significantly improved disease-free survival compared with placebo in patients at high risk of recurrence after radical surgery, establishing an important antibody-based treatment option in the postoperative setting [58]. Similarly, the phase III AMBASSADOR trial demonstrated significantly longer disease-free survival with adjuvant pembrolizumab than with observation in patients with high-risk muscle-invasive urothelial carcinoma [59].

More recently, peri-operative ICI therapy combined with chemotherapy has produced clinically meaningful survival benefits. In the phase III NIAGARA trial, peri-operative durvalumab combined with neoadjuvant gemcitabine–cisplatin followed by radical cystectomy and adjuvant durvalumab significantly improved event-free survival and overall survival compared with neoadjuvant chemotherapy and surgery alone. At 24 months, event-free survival was 67.8% versus 59.8% and overall survival was 82.2% versus 75.2%, respectively [60].

The most important recent development for ADC-based therapy is the emergence of peri-operative enfortumab vedotin (EV) plus pembrolizumab. In the phase III KEYNOTE-905/EV-303 trial, peri-operative EV plus pembrolizumab significantly improved event-free and overall survival in cisplatin-ineligible or cisplatin-declining patients with MIBC compared with radical cystectomy and pelvic lymph-node dissection alone. The trial therefore provides prospective evidence that an ADC–ICI combination can deliver clinically meaningful benefit in a potentially curative setting [61].

Importantly, the subsequent KEYNOTE-B15/EV-304 trial extended these findings to cisplatin-eligible patients. Peri-operative EV plus pembrolizumab significantly improved event-free survival, overall survival, and pCR compared with neoadjuvant cisplatin–gemcitabine followed by surgery. At 2 years, event-free survival was 79.4% versus 66.2%, overall survival was 86.9% versus 81.3%, and pCR was 55.8% versus 32.5%, respectively. However, grade 3 or higher adverse events were more frequent with EV plus pembrolizumab, highlighting the importance of careful patient selection and toxicity management when antibody-based combinations are moved into curative-intent settings [62].

Collectively, these findings indicate a fundamental shift in the clinical development of antibody-mediated therapies in UCC. Rather than being restricted to treatment-refractory metastatic disease, ICIs and ADC–ICI combinations are increasingly being evaluated across the peri-operative continuum, including neoadjuvant, adjuvant, and peri-operative strategies. This transition raises several important questions regarding optimal patient selection, treatment duration, integration with radical surgery, and the role of pathological response and molecular residual disease as treatment-guiding biomarkers. In particular, whether peri-operative ADC–ICI therapy can facilitate treatment de-escalation or organ-preservation strategies in selected patients remains an important area for future investigation.

## 5. Resistance Development in Antibody-Mediated Therapies

Despite the clinical advances achieved with antibody-mediated therapies, resistance remains a major unmet need in UCC. Both primary (innate) resistance, in which tumors fail to respond from the outset, and acquired resistance, in which initially responsive tumors later relapse, limit therapeutic efficacy and durability of response.

### 5.1. Mechanisms of Resistance

1. Antigen loss or downregulation: In UCC, heterogeneous expression of Nectin-4, Trop-2, and HER2 may influence ADC sensitivity [26,63,64]. Notably, reduced or absent membranous Nectin-4 expression in metastatic lesions has been associated with decreased benefit from enfortumab vedotin [65].
2. Impaired payload delivery: Even when antigen binding is intact, cytotoxic payloads may not be effectively delivered due to defective endocytosis, altered lysosomal processing, or upregulation of drug efflux transporters [66,67,68].
3. Tumor Heterogeneity: Intratumoral and interlesional heterogeneity may generate subclones with variable ADC target expression or intrinsic differences in drug sensitivity, allowing antigen-low or resistant subclones to persist under therapeutic pressure and contribute to disease progression [69].
4. Immunosuppressive Tumor Microenvironment (TME): Resistance to ICIs, and potentially to ADC–ICI combinations, may be promoted by an immunosuppressive TME characterized by T-cell exclusion, poor cytotoxic T-cell infiltration, TGF-β–associated stromal signaling, and suppressive immune populations such as Tregs and MDSCs, thereby limiting immune-mediated tumor killing [23,70,71].
5. Adaptive and Compensatory Pathways: Tumor cells may evade antibody-mediated therapy by activating compensatory receptor tyrosine kinase signaling or alternative survival pathways. Although direct evidence in UCC remains limited, HER2-directed ADC studies in other solid tumors suggest that EGFR- or HER3-related signaling may influence ADC internalization, efficacy, and resistance [72,73].

Among them, low or absent target antigen expression, deficient immune infiltration, or pre-existing immunosuppressive tumor microenvironments are related to Primary. Others are related to acquired resistance.

### 5.2. Clinical Implications

Resistance is associated with shortened duration of response, limited progression-free and overall survival, and decreased efficacy of sequential therapies. This highlights an urgent need for:

- Biomarker-guided patient selection to identify individuals likely to respond;
- Rational combination therapies designed to overcome or delay resistance;
- Real-time monitoring of ctDNA;
- Such strategies are essential to prolong response and improve patient outcomes.

## 6. Biomarkers and Patient Selection

Optimizing antibody-mediated therapies in urothelial carcinoma (UCC) requires identification of predictive biomarkers to enhance efficacy while minimizing unnecessary toxicity. Both immune checkpoint inhibitors (ICIs) and antibody–drug conjugates (ADCs) benefit from biomarker-guided patient selection, though challenges remain in standardization and clinical application.

### 6.1. Biomarkers for ICIs

Predictive biomarkers are essential for selecting patients most likely to benefit from ICIs.

- PD-L1 expression is the most extensively studied biomarker. Tumors or immune cells with high PD-L1 expression generally exhibit enhanced T-cell activation and improved response rates. Limitations include inter-assay variability, intratumoral heterogeneity, and dynamic changes over time, which reduce its predictive reliability [74,75].
- Tumor mutational burden (TMB) provides complementary information: high TMB increases neoantigen load, potentially enhancing immunogenicity and responsiveness to PD-1/PD-L1 blockade. Lack of standardized cutoffs and incomplete capture of all responders limits its standalone utility [76,77].
- Emerging biomarkers include T-cell-inflamed/immune gene signatures, microsatellite instability (MSI-H/dMMR), and circulating tumor DNA (ctDNA). These can reflect pre-existing immune activation, identify high mutational load, or provide dynamic monitoring of tumor burden and emerging resistance, supporting more precise, personalized ICI strategies [78,79,80].

Table 3 summarizes key predictive biomarkers for immune checkpoint inhibitor (ICI) therapy in urothelial carcinoma. Each biomarker is presented with its mechanistic rationale and known limitations. PD-L1 expression and TMB are the most widely studied, whereas T-cell-inflamed gene signatures and ctDNA offer complementary or dynamic predictive value. MSI-H/dMMR is rare but indicates high tumor immunogenicity. Collectively, these biomarkers support patient selection, though further validation and standardization are needed for optimal clinical use.

### 6.2. Biomarkers for Antibody–Drug Conjugates (ADCs)

The efficacy of ADCs in urothelial carcinoma (UCC) is influenced by a combination of on-target antigen expression, tumor-intrinsic biology, and tumor microenvironmental factors.

**Enfortumab Vedotin (EV):**

- Primary biomarker: Nectin-4 expression and membranous localization, essential for ADC binding, internalization, and payload delivery [25].
- Resistance mechanisms: Downregulation or loss of Nectin-4 reduces surface binding and internalization [65].
- Tumor-intrinsic factors: Alterations in CDKN2A/CDKN2B and TSC1 may modulate sensitivity indirectly via cell-cycle and mTOR pathways, reflecting broader susceptibility rather than direct ADC targeting [81,82].
- Clinical relevance: Most data are preclinical or retrospective; prospective validation is limited.

**Sacituzumab Govitecan (SG):**

- Primary biomarker: Trop-2 expression facilitates ADC internalization and SN-38 delivery [83].
- Considerations: Clinical responses occur across a broad range of Trop-2 expression; no validated cutoff exists [63].
- Limitations: Tumor heterogeneity may limit uniform ADC uptake, although the high drug–antibody ratio and membrane-permeable payload enable a partial bystander effect [84].
- Additional factors: DNA damage response (DDR) defects may increase SN-38 sensitivity, while prior topoisomerase I inhibitor exposure may reduce response. ADC-induced immunogenic cell death may enhance efficacy in combination with ICIs [85].

**Disitamab Vedotin (DV):**

- Primary biomarker: HER2 expression by immunohistochemistry (IHC) is the most clinically relevant predictor; activity is observed even in HER2-low (IHC 1+) tumors due to a potent bystander effect mediated by MMAE [86].
- Secondary biomarkers: HER2 amplification (FISH) correlates with protein expression but is not mandatory for response [64].
- Resistance mechanisms: Tumor HER2 heterogeneity, HER2 shedding, receptor downregulation, and upregulation of drug efflux transporters (e.g., MDR1) can limit ADC activity [82,87].
- Combination potential: ADC-induced immunogenic cell death may synergize with PD-1/PD-L1 blockade, providing rationale for combination therapies [88].

**Summary:**

While on-target antigen expression is necessary, ADC efficacy in UCC is determined by a complex convergence of antigen density, tumor-intrinsic signaling, payload susceptibility, and immune microenvironmental factors. This multifaceted requirement highlights an urgent need for prospective validation of these biomarkers to optimize patient selection and sequencing strategies.

Table 4 summarizes key biomarkers associated with response and resistance to ADCs in UCC. On-target biomarkers are required for ADC activity, predictive biomarkers influence sensitivity, resistance biomarkers reduce efficacy, and off-target/bystander effects reflect tumor-intrinsic modulation. Immune microenvironment markers indicate potential synergy with ICIs. This table is intended to provide a mechanistic framework for understanding factors influencing ADC efficacy and resistance and to guide biomarker-driven patient selection and combination therapy strategies. Notes emphasize assay limitations, heterogeneity, and validation status.

## 7. Combination Therapies

### 7.1. Rationale for ADC-ICI Combination Therapy

ADC-ICI combinations are designed to integrate targeted tumor-cell killing with immune activation. ADC-mediated cytotoxicity may promote antigen release and immunogenic cell death, while ICIs can restore antitumor T-cell activity and counter adaptive immune suppression within the tumor microenvironment. Here is the rationale broken down by mechanisms:

#### 7.1.1. Synergistic Effects of Spatial and Temporal Mechanisms

ADCs provide spatially targeted cytotoxicity by delivering payloads to antigen-expressing tumor cells, whereas ICIs enhance systemic antitumor immunity by restoring T-cell activity. ADC-mediated tumor-cell death may promote antigen release and immunogenic cell death, thereby increasing opportunities for immune recognition [89,90]. In this context, ICIs may help sustain and amplify antitumor T-cell responses, providing a mechanistic rationale for ADC–ICI synergy.

#### 7.1.2. Overcoming the Limitations of the Bystander Effect

The bystander effect allows ADCs to kill neighboring tumor cells with low or heterogeneous target-antigen expression, but this effect may be limited by payload diffusion, tumor architecture, and the extent of antigen-positive cell distribution [91,92,93]. ADC–ICI combination therapy may help overcome these limitations by linking ADC-mediated tumor-cell death and antigen release with T-cell-mediated antitumor immunity, thereby broadening tumor control beyond direct ADC-targeted cells [94,95].

#### 7.1.3. Counteracting Treatment-Induced Adaptive Immune Suppression

ADC-mediated tumor-cell death may promote antigen release and immunogenic cell death [96], but cytotoxic stress can also induce counter-regulatory immune-suppressive pathways, including PD-L1 upregulation or inhibitory damage-associated signals in some contexts [97]. Combining ADCs with ICIs may counter PD-1/PD-L1-mediated adaptive immune resistance and enhance T-cell-mediated tumor killing [94,96].

#### 7.1.4. Transition from Transient Response to Durable Benefit

Although ADCs can induce rapid tumor responses, resistance and relapse may limit the durability of benefit. Importantly, early tumor responses do not necessarily translate into durable clinical benefit, as illustrated by the failure of SG to demonstrate a significant survival benefit in the phase III TROPiCS-04 trial [54]. ADC–ICI combination therapy may extend this benefit by coupling ADC-mediated tumor-cell killing with ICI-driven restoration of antitumor T-cell activity and adaptive immune responses [94,97].

### 7.2. Clinical Impact of ADC–ICI Combination Therapies

Combination strategies with ICIs enhance efficacy considerably:

- EV + pembrolizumab in the first-line metastatic setting achieves ORRs of 68%, median PFS of 12.3 months, and median OS exceeding 23 months [98,99].
- SG + pembrolizumab after platinum-based chemotherapy has shown ORRs of 34–41%, with median PFS around 5–6 months and a median OS of 12.7 months in the phase II TROPHY-U-01 cohort 3 study [100]. However, these findings should be interpreted cautiously because the study was single-arm and exploratory, and the subsequent phase III TROPiCS-04 trial failed to demonstrate a significant OS benefit for SG monotherapy compared with physician’s choice chemotherapy, ultimately leading to withdrawal of the FDA-approved urothelial carcinoma indication for SG [54,55].
- DV + toripalimab in HER2-positive patients demonstrates ORRs approaching 76% and median OS exceeding 30 months [101].

These results highlight a synergistic interaction between ADC-mediated tumor cytotoxicity and ICI-driven immune activation, resulting in higher response rates, deeper remissions, and prolonged survival. While SG + pembrolizumab shows preliminary activity, the magnitude of benefit appears less pronounced than with EV- or DV-based combinations (Table 5). In addition, the withdrawal of the SG urothelial carcinoma indication following the negative phase III TROPiCS-04 trial underscores the need to distinguish encouraging response rates observed in early-phase combination studies from confirmed survival benefit in randomized trials [54,55,100].

Overall, this comparative analysis supports a paradigm shift in UCC management: single-agent ADCs offer substantial benefit in refractory disease, whereas ADC–ICI combinations consistently achieve superior clinical outcomes, particularly in earlier lines of therapy. Rationally designed combinations guided by tumor antigen expression and immune contexture are poised to become foundational components of treatment for molecularly selected patients with UCC.

## 8. Real-Time Monitoring of ctDNA

### 8.1. Rationale for Real-Time Monitoring of ctDNA

#### 8.1.1. Early Detection of Resistance via ctDNA

Conventional imaging remains essential for response assessment, but serial ctDNA monitoring may detect molecular progression or relapse before radiographic progression in selected patients with UCC [102,103]. Changes in ctDNA levels and emerging genomic alterations can provide early information on tumor dynamics and evolving resistance [104].

#### 8.1.2. Tracking Clonal Evolution via ctDNA for Adaptive Treatment Strategies

Because tumor genomic profiles can evolve over time, serial ctDNA monitoring provides a minimally invasive approach to assess dynamic changes in tumor burden and genomic alterations [104,105]. In UCC, serial ctDNA analysis may help track emerging resistant clones and support treatment adaptation or clinical trial selection [104].

#### 8.1.3. Tracking Bypass Escape Routes to Optimize ADC and ICI Therapies

Resistance to antibody-mediated therapies in UCC may involve changes in target-antigen expression, immune escape mechanisms, or activation of alternative oncogenic pathways [106]. Broad-panel ctDNA-based NGS can noninvasively monitor evolving genomic alterations and may identify potentially actionable changes, such as FGFR2/3 alterations, during treatment [104,107,108].

### 8.2. Clinical Results for Real-Time Monitoring of ctDNA

Unlike other types of cancer, research on real-time monitoring of ctDNA to overcome resistance is still in the clinical trial stage at the UCC, and the published research results are as follows. In a multicenter retrospective real-world analysis of 110 patients with advanced urothelial carcinoma treated with enfortumab vedotin with or without pembrolizumab, longitudinal tumor-informed ctDNA monitoring using Signatera was strongly associated with clinical outcomes. Persistent ctDNA negativity between weeks 4 and 24 was associated with durable PFS (HR 0.05) and OS (HR 0.046), whereas on-treatment ctDNA positivity correlated with inferior survival outcomes [109].

## 9. Current Challenges and Unmet Needs

Despite the rapid advances in antibody-mediated therapies for UCC, several challenges limit their optimal clinical implementation.

### 9.1. Therapeutic Sequencing

The optimal sequence of ICIs, ADCs, and chemotherapy has not yet been clearly defined. The emergence of enfortumab vedotin plus pembrolizumab (EV+pembrolizumab) as a preferred first-line treatment has substantially changed the treatment landscape [98], with many patients now remaining platinum-naïve at disease progression. In EV-302, platinum-based chemotherapy was the most commonly used subsequent treatment after EV+pembrolizumab; however, the lack of prospective data on treatment sequencing strategies complicates treatment decisions.

Potential strategies to overcome this include:

- Biomarker-based treatment sequencing: Using PD-L1, antigen expression, or mutation profiling to determine treatment sequence. Biomarker-directed therapies, including erdafitinib for susceptible FGFR2/3-altered tumors and HER2-directed therapies for selected HER2-positive tumors, may provide alternative treatment options after progression on EV + pembrolizumab [110].

Selection of subsequent ADCs: The optimal use and sequencing of subsequent ADCs after prior EV exposure remain uncertain because resistance may arise through target downregulation, altered internalization, or payload-related mechanisms. The phase III TROPiCS-04 trial did not demonstrate a significant overall survival or progression-free survival benefit for sacituzumab govitecan over physician’s choice chemotherapy, highlighting the need for prospective evaluation of ADC sequencing [54].

- Combination therapy: Co-administration of ADCs and ICIs can reduce uncertainty in treatment sequencing with careful toxicity monitoring.
- Dynamic response monitoring: Using liquid biopsy and imaging analysis to adjust treatment regimens based on the development of resistance.

Given the absence of prospective evidence defining the optimal post-EV+pembrolizumab treatment sequence, subsequent therapy should be individualized according to prior treatment exposure, residual toxicity, performance status, and biomarker profile. Clinical trial enrollment should be strongly considered whenever feasible, particularly for patients progressing after EV+pembrolizumab.

### 9.2. Limited Long-Term Survival Data

While pivotal trials demonstrate encouraging short-term efficacy, long-term survival data remain sparse. Most studies report median follow-up durations of 12–24 months, which may not capture:

- Durability of response: Although some patients achieve durable tumor control with ADC- or ICI-based therapies, many still experience disease progression within 1–2 years, particularly in later-line settings [98,99]. Because several pivotal ADC–ICI studies have relatively limited follow-up, the clinical and molecular features that define long-term responders remain incompletely understood [98,111].
- Late-onset toxicities: Immune-related adverse events (irAEs) from ICIs may emerge months or even years after therapy initiation or discontinuation [112]. ADC-related toxicities, including peripheral neuropathy, hepatotoxicity, and cumulative organ dysfunction, may also develop over time [30,52,113].
- Combination regimens: ADC–ICI combinations may enhance antitumor efficacy, but the long-term safety and tolerability of these regimens remain largely undefined, raising concerns about chronic or cumulative toxicity.
- Overall, the lack of extended follow-up restricts the ability to predict long-term outcomes, emphasizing the need for prospective, long-term studies and real-world evidence.

### 9.3. Biomarker Limitations

- **PD-L1 expression**: While higher PD-L1 levels can correlate with response to ICIs, assay variability, intratumoral heterogeneity, and dynamic expression limit its predictive reliability [44,114].
- **Tumor mutational burden (TMB) and gene signatures:** High TMB or T-cell-inflamed gene expression may indicate increased immunogenicity, but no standardized cutoffs exist, and some patients with low TMB still respond [115,116].
- **Antigen expression for ADCs**: Targets such as Nectin-4, Trop-2, and HER2 provide the biological basis for ADC therapy in UCC, yet heterogeneous or low-level expression, as well as antigen downregulation, may reduce efficacy [12,69,117]. Determining clinically meaningful thresholds for positivity remains unresolved.
- Misclassification of patients may lead to unnecessary exposure to toxic therapies, increased healthcare costs, and missed opportunities for alternative strategies.

### 9.4. Tumor Heterogeneity

- Intra- and intertumoral heterogeneity complicates patient selection, as different tumor regions may exhibit variable antigen expression or immune infiltration [39,69].
- Resistant subclones may be present from the outset, leading to primary resistance, even in biomarker-positive tumors [118,119].

### 9.5. Dynamic Disease Evolution

- Antigen levels, immune contexture, and molecular pathways can change over time, especially after prior therapies (e.g., platinum chemotherapy or ICIs) [65,106,120].
- Single-timepoint biomarker assessments may not accurately reflect current tumor biology, reducing predictive accuracy [104].

## 10. Future Directions

### 10.1. Ongoing and Emerging Clinical Development

#### 10.1.1. Expansion into Earlier Disease Settings

The clinical success of antibody-mediated therapies in advanced UCC is rapidly driving their evaluation in earlier disease settings. The phase III KEYNOTE-905/EV-303 trial demonstrated significant event-free and overall survival benefits with perioperative enfortumab vedotin (EV) plus pembrolizumab in patients with muscle-invasive bladder cancer who were cisplatin-ineligible or declined cisplatin, supporting the potential role of ADC–ICI combinations in curative-intent treatment [61]. Ongoing studies are further evaluating ADC-based approaches in neoadjuvant and perioperative settings, including upper-tract urothelial carcinoma [121]. Future studies should determine whether these strategies can improve pathologic response, recurrence-free survival, and ultimately cure rates while maintaining an acceptable long-term toxicity profile.

#### 10.1.2. Development of Next-Generation ADCs and Novel Targets

The therapeutic landscape is expanding beyond currently established ADCs toward next-generation platforms with optimized antibody specificity, linker stability, payload selection, drug-to-antibody ratio, and bystander effects. Increasingly, ADC development is also exploring payload diversification and more complex molecular architectures, including novel cytotoxic payloads, dual-payload ADCs, and bispecific or dual-targeting ADCs designed to address tumor heterogeneity and treatment resistance [122,123,124]. These approaches seek to improve the therapeutic index, broaden activity across tumors with heterogeneous antigen expression, and overcome resistance mechanisms associated with conventional single-target, single-payload ADCs.

Bulumtatug fuvedotin (BFv; 9MW2821), a next-generation Nectin-4-directed ADC, has demonstrated encouraging activity in advanced UCC as monotherapy and in combination with toripalimab [46,47,48]. Randomized phase III studies are evaluating BFv versus chemotherapy in previously treated advanced UCC and BFv plus toripalimab versus standard chemotherapy in the first-line setting [125,126]. Key clinical development programs involving next-generation ADCs and emerging antibody targets in UCC are summarized in Table 6.

In parallel, development is expanding toward additional tumor-associated antigens, including HER3, CDH6, SLITRK6, and B7-H3. The EGFR×HER3 bispecific ADC izalontamab brengitecan is being evaluated in metastatic UCC after immunotherapy [127]. CDH6 is another emerging ADC target, with raludotatug deruxtecan demonstrating CDH6-dependent internalization and antitumor activity in preclinical models [49].

SLITRK6 also represents a urothelial cancer-associated target of particular interest. The investigational ADC AGS15E (sirtatumab vedotin), which delivers MMAE to SLITRK6-expressing tumor cells, demonstrated clinical activity in a phase I study of heavily pretreated metastatic UCC, providing proof of concept for SLITRK6-directed therapy [35]. B7-H3 is another emerging tumor-associated antigen, and ifinatamab deruxtecan has demonstrated early clinical activity in a phase 1/2 pan-tumor study that included patients with bladder cancer [37]. Collectively, these approaches may broaden the population eligible for ADC therapy and provide alternative therapeutic strategies for tumors that lack sufficient expression of, or develop resistance to, currently established ADC targets. Importantly, the increasing diversity of ADC targets and molecular architectures may enable more individualized selection of ADCs according to antigen expression, tumor heterogeneity, and specific mechanisms of treatment resistance.

#### 10.1.3. Rational Combination Strategies

Combination strategies represent an important direction of future antibody-mediated therapy in UCC. Beyond the established EV–pembrolizumab regimen, next-generation ADC–ICI combinations, including BFv plus toripalimab and disitamab vedotin plus pembrolizumab, are being evaluated in randomized studies [126,128]. Rather than reiterating the mechanistic rationale for ADC–ICI synergy described above, ongoing clinical development is increasingly exploring biologically informed multimodal strategies that target complementary aspects of tumor biology.

One emerging approach is the combination of two ADCs directed against distinct tumor-associated antigens. The phase I Double Antibody Drug Conjugate (DAD) study evaluated sacituzumab govitecan plus enfortumab vedotin, thereby combining TROP-2- and Nectin-4-directed ADCs with different cytotoxic payloads, and provided clinical proof of concept for a dual-ADC strategy in metastatic UCC [129]. The combination produced an objective response rate of 70% in the evaluable population, although grade ≥3 adverse events were frequent, highlighting the need to define the therapeutic window of dual-ADC regimens. Such approaches may increase tumor-cell coverage in tumors characterized by heterogeneous antigen expression and provide complementary mechanisms of cytotoxicity. However, optimal patient selection, treatment sequencing, pharmacologic interactions, and cumulative toxicity remain to be established.

Beyond combinations of separate ADCs, next-generation ADC engineering is also expanding toward more complex molecular architectures. These include single ADC molecules incorporating two distinct cytotoxic payloads, as well as bispecific or dual-targeting ADCs capable of recognizing two different tumor-associated antigens [122,123,124]. Dual-payload ADCs are being investigated as a means of delivering cytotoxic agents with complementary mechanisms of action within the same molecular construct, potentially improving activity against heterogeneous tumor cell populations and overcoming mechanisms of acquired resistance [122,123]. Bispecific ADCs, in turn, may simultaneously engage two distinct antigens or epitopes, potentially increasing tumor-cell coverage, improving target selectivity, or facilitating internalization in tumors with heterogeneous antigen expression [122,124]. These approaches represent an evolution from conventional single-target, single-payload ADCs toward more sophisticated platforms designed to address the biological complexity of treatment-resistant tumors.

Triplet strategies combining an ADC, an ICI, and a molecularly targeted agent represent a further extension of this biologically informed approach. In FGFR3-altered UCC, vepugratinib is being evaluated in the phase I FORAGER-1 study and in combination with EV and pembrolizumab in the randomized phase III FORAGER-2 study [130,131]. In FORAGER-2, vepugratinib is being evaluated in combination with EV and pembrolizumab versus EV and pembrolizumab alone in previously untreated locally advanced or metastatic UCC harboring an activating FGFR3 alteration [131]. By simultaneously targeting tumor-cell surface antigens, immune signaling, and oncogenic driver pathways, these regimens seek to address complementary mechanisms of tumor growth and treatment resistance. Such multimodal strategies may be particularly relevant for molecularly defined subgroups in which a targetable oncogenic alteration coexists with an actionable tumor-associated antigen and an immunologically permissive tumor microenvironment.

Overall, future combination strategies should move beyond empirical treatment intensification toward biomarker-guided approaches that exploit complementary vulnerabilities while minimizing cumulative toxicity. Randomized studies will be needed to determine whether dual-ADC combinations, next-generation ADC architectures, and ADC–ICI-targeted therapy triplets provide clinically meaningful improvements in survival, durability of response, or resistance control compared with established treatment strategies. Importantly, increasing therapeutic complexity will require careful consideration of treatment sequencing, pharmacologic interactions, cumulative toxicity, and predictive biomarkers to ensure that greater molecular and therapeutic breadth translates into meaningful clinical benefit.

**Table 6:** Selected ongoing clinical development of antibody-mediated therapies in urothelial carcinoma.

Therapeutic Strategy	Target	Agent/Combination	Disease Setting	Phase	Clinical Trial
Perioperative ADC–ICI	Nectin-4 + PD-1	EV + pembrolizumab	MIBC, cisplatin-ineligible or cisplatin-declining	III	KEYNOTE-905/EV-303 [61]
Neoadjuvant ADC–ICI	Nectin-4 + PD-1	EV + pembrolizumab	Localized/locally advanced UTUC	II	Ongoing [121]
HER2-directed ADC–ICI	HER2 + PD-1	DV + pembrolizumab	Previously untreated HER2-expressing advanced UCC	III	NCT05911295 [128]
Next-generation Nectin-4 ADC	Nectin-4	BFv (9MW2821)	Previously treated advanced UCC	III	NCT06196736 [125]
Nectin-4 ADC–ICI	Nectin-4 + PD-1	BFv + toripalimab	First-line locally advanced/metastatic UCC	III	NCT06592326 [126]
TROP-2-directed ADC	TROP-2	Datopotamab deruxtecan + platinum chemotherapy	Locally advanced/metastatic UCC	III	NCT07129993 [132]

Abbreviations: ADC, antibody–drug conjugate; ICI, immune checkpoint inhibitor.

### 10.2. Biomarker Development, Resistance Mechanisms, and Treatment Sequencing

#### 10.2.1. Biomarker-Guided and Multiparametric Treatment Selection

A major future priority is the development of robust biomarkers for selecting the optimal antibody-mediated therapy. As discussed above, PD-L1 expression, TMB, and individual antigen expression have important limitations when used as single biomarkers. Future strategies should integrate multiple dimensions of tumor biology, including antigen density and heterogeneity, genomic alterations, immune-related signatures, and characteristics of the tumor microenvironment [133].

This approach may enable treatment selection according to the biological characteristics of individual tumors rather than relying on a single predefined biomarker. Recent analyses of Nectin-4, HER2, and TROP-2 expression have demonstrated substantial intertumoral and intratumoral heterogeneity, supporting the need to consider both antigen expression level and spatial distribution when selecting ADC-based therapies [134]. In particular, transcriptomic and multiparametric approaches may help identify complementary or mutually exclusive ADC target profiles and guide selection among Nectin-4-, HER2-, TROP-2-, and emerging target-directed therapies. Recent clinical development increasingly supports this shift toward precision selection of ADC platforms rather than treating ADCs as a single therapeutic class.

#### 10.2.2. Resistance Mechanisms and Treatment Sequencing

As ADCs move into earlier lines of therapy, resistance after prior ADC exposure will become an increasingly important clinical problem. Future treatment strategies should account for mechanisms of acquired ADC resistance, including target-antigen loss or downregulation [65], impaired internalization, intracellular trafficking, or lysosomal processing [66,67,68], and upregulation of drug-efflux transporters or payload-specific resistance [68,82].

Rather than assuming that sequential use of different ADCs will necessarily overcome resistance, prospective studies should evaluate whether switching the target, antibody, linker, or payload can provide meaningful clinical benefit. Similarly, the optimal sequencing of ADCs, ICIs, chemotherapy, and molecularly targeted therapies remains to be established. Randomized trials and translational studies incorporating serial tumor and liquid-biopsy analyses will be important for defining rational treatment sequences.

### 10.3. Precision and Adaptive Treatment Strategies

#### 10.3.1. Integration of Real-Time Molecular Monitoring

Serial ctDNA analysis may provide an important tool for future adaptive treatment strategies. Although ctDNA monitoring remains investigational in UCC, recent multicenter real-world data in patients treated with EV ± pembrolizumab demonstrated a strong association between longitudinal ctDNA dynamics and clinical outcomes. On-treatment ctDNA positivity was consistently associated with inferior PFS and OS, whereas early ctDNA clearance was associated with radiographic response [135].

Future prospective studies should determine whether ctDNA can move beyond a prognostic biomarker to become a predictive and treatment-guiding tool. Integration of ctDNA with radiographic assessment, target-antigen expression, and molecular profiling could potentially enable earlier recognition of treatment failure and selection of subsequent therapy before overt radiographic progression. However, such strategies require prospective validation before routine clinical implementation.

#### 10.3.2. Toward Personalized and Adaptive Antibody-Based Treatment

Collectively, the next phase of antibody-mediated therapy in UCC should move beyond the development of increasingly potent agents toward biologically informed and adaptive treatment strategies. Integration of next-generation ADCs, novel tumor-associated antigens, rational ADC–ICI combinations, multiparametric biomarkers, and longitudinal molecular monitoring may enable treatment selection and modification according to the evolving biology of individual tumors. Treatment rechallenge may also represent a potential strategy for selected patients who previously derived substantial benefit from antibody-based therapy. In a multicenter post hoc analysis of patients with advanced or metastatic UCC who had previously responded to pembrolizumab-based therapy or completed two years of treatment, pembrolizumab retreatment produced an objective response in 41% of patients, with a median progression-free survival of 9.5 months and median overall survival of 25.7 months from retreatment initiation [136]. These findings suggest that rechallenge may be clinically relevant in carefully selected patients, particularly those with a prior durable response, although prospective studies are needed to define the optimal patient population and treatment interval.

Future clinical development should therefore focus not only on maximizing response rates but also on identifying patients most likely to achieve durable benefit, defining rational treatment sequences and rechallenge strategies, and minimizing cumulative toxicity. Such an approach may ultimately transform antibody-mediated therapy from a predominantly drug-centered strategy into a more precise, biomarker-driven, and dynamically personalized treatment paradigm.

## 11. Conclusions

Antibody-mediated therapies have substantially reshaped the treatment landscape of urothelial carcinoma, with immune checkpoint inhibitors and antibody–drug conjugates providing clinically meaningful benefits across different stages of disease. The development of enfortumab vedotin, sacituzumab govitecan, and HER2-directed ADCs has established the therapeutic potential of antibody-based approaches, while next-generation ADCs and novel tumor-associated targets are further expanding the therapeutic landscape.

Future progress will depend not only on the development of increasingly potent antibody-based agents but also on improved patient selection, rational treatment sequencing, and effective management of acquired resistance. Integration of multiparametric biomarkers, novel ADC platforms, ADC–ICI combinations, and longitudinal molecular monitoring may enable more individualized and adaptive treatment strategies. Ultimately, prospective randomized trials and long-term clinical and translational studies will be essential to determine which antibody-mediated approaches provide durable survival benefit while minimizing cumulative toxicity.

## Figures and Tables

**Table 1:** Summary of basic structural comparison.

Category	Enfortumab Vedotin (EV)	Sacituzumab Govitecan (SG)	Disitamab Vedotin (DV)
Target antigen	Nectin-4	Trop-2	HER2
Antibody	Fully human IgG1	Humanized IgG1	Humanized IgG1
Linker	Protease-cleavable (Val-Cit)	Hydrolysable (CL2A)	Protease-cleavable (Val-Cit)
Payload	MMAE (microtubule inhibitor)	SN-38 (Topoisomerase I inhibitor)	MMAE
Average DAR	~3–4	~7–8 (high)	~3–4
Bystander effect	Moderate	Strong	Moderate

Abbreviations: DAR, drug-to-antibody ratio; EV, enfortumab vedotin; SG, sacituzumab govitecan; DV, disitamab vedotin; MMAE, monomethyl auristatin E; SN-38, 7-ethyl-10-hydroxycamptothecin.

**Table 2:** The clinical activity and survival outcomes of major antibody–drug conjugates (ADCs) in urothelial carcinoma (UCC).

Therapy	Target	Treatment Setting	ORR	Median PFS	Median OS	Clinical Significance
EV (Enfortumab Vedotin)	Nectin-4	Post-treatment (including platinum-refractory)	40–50%	5.8–6.5 months	11.7–12.8 months	Standard option in later-line therapy; durable responses possible
SG (Sacituzumab Govitecan)	Trop-2	Later-line	23–32%	4.23–5.8 months	10.3–13.5 months	Later-line option for Trop-2–positive disease; monotherapy activity
DV (Disitamab Vedotin)	HER2	Later-line	50–55%	5–6 months	14 months	Option for HER2-positive urothelial cancer

Abbreviations: ORR, objective response rate; PFS, progression-free survival; OS, overall survival.

**Table 3:** Predictive biomarkers for response to immune checkpoint inhibitors in urothelial carcinoma: mechanisms and limitations.

Biomarker	Mechanism/Rationale	Limitations
PD-L1 expression	Higher expression on tumor or immune cells predicts enhanced T-cell activation upon PD-1/PD-L1 blockade	Assay variability, intratumoral heterogeneity, dynamic expression
Tumor Mutational Burden (TMB)	High TMB → more neoantigens → increased immunogenicity	No standardized cutoff; may not capture all responders
T-cell-inflamed/immune gene signatures	Reflect pre-existing immune activation within tumor microenvironment	Requires complex transcriptomic profiling; limited prospective validation
Microsatellite instability/Mismatch repair deficiency (MSI-H/dMMR)	High mutational load and neoantigen formation	Low prevalence in urothelial carcinoma
Circulating tumor DNA (ctDNA)	Dynamic monitoring of tumor burden and emerging resistance	Still investigational; lacks standardized thresholds

**Table 4:** Biomarkers of response and resistance to antibody–drug conjugates in urothelial carcinoma: mechanisms.

ADC	Biomarker	Type	Mechanistic Rationale	Notes
Enfortumab Vedotin (EV)	Nectin-4 expression/membranous localization	On-target	Required for ADC binding, internalization, and payload delivery	Membranous vs. cytoplasmic distinction often unclear
EV	Nectin-4 copy number/density	On-target	Higher copy number/membrane density may enhance ADC uptake	Observed in vitro and xenograft models; clinical validation pending
EV	Nectin-4 downregulation/loss	Resistance/On-target	Reduced surface antigen limits ADC binding and internalization	Observed in resistant tumors; limited prospective validation
EV	CDKN2A/CDKN2B alterations	Off-target/Bystander effect	Cell-cycle pathway alterations may influence tumor proliferation and sensitivity indirectly	Associated with shorter PFS in EV-treated patients; reflects tumor biology rather than direct ADC binding
EV	TSC1 mutations	Off-target/Bystander effect	mTOR pathway mutation may increase sensitivity to cytotoxic stress	Linked to higher ORR; likely represents tumor-intrinsic susceptibility
Sacituzumab Govitecan (SG)	Trop-2 expression (IHC)	On-target	ADC binds Trop-2; higher expression enhances internalization and SN-38 delivery	Responses observed across a wide range of Trop-2 expression; no validated cutoff
SG	Trop-2 heterogeneity	On-target (negative)	Heterogeneous expression may limit uniform ADC uptake	High drug–antibody ratio and bystander effect may partially overcome heterogeneity
SG	DNA damage response (DDR) defects	Predictive (exploratory)	Impaired DNA repair may increase SN-38 sensitivity	Hypothesis-generating; not clinically validated
SG	Prior topoisomerase I inhibitor exposure	Predictive (negative)	Potential cross-resistance to SN-38	Clinical relevance in urothelial carcinoma remains unclear
SG	Immune microenvironment (PD-L1, TILs)	Predictive (combination)	SN-38-induced immunogenic cell death may synergize with ICIs	Relevant for SG + pembrolizumab combinations
Disitamab Vedotin (DV)	HER2 expression (IHC)	Predictive/On-target	HER2-directed ADC; higher HER2 expression increases binding, internalization, and payload delivery	Activity observed even in HER2-low (IHC 1+) tumors, broader than conventional HER2-targeted therapies
DV	HER2 amplification (FISH)	Predictive/On-target	Gene amplification correlates with increased HER2 protein expression and ADC uptake	Not mandatory for response; IHC more clinically relevant than amplification status
DV	HER2-low expression	Predictive/On-target	Strong bystander killing effect via membrane-permeable MMAE payload	Expands treatable population beyond conventional HER2-high tumors
DV	Tumor HER2 heterogeneity	Predictive (negative)	Heterogeneous HER2 expression may limit uniform ADC delivery	Bystander effect may partially overcome heterogeneity
DV	Drug efflux transporter expression (e.g., MDR1)	Resistance	Upregulation may reduce intracellular MMAE concentration	Suggested mechanism of acquired resistance
DV	HER2 shedding/receptor downregulation	Resistance	Reduced surface HER2 decreases ADC binding	Observed in resistance models
DV	Immune microenvironment (PD-L1, TILs)	Predictive (combination)	ADC-induced immunogenic cell death may synergize with PD-1/PD-L1 blockade	Relevant for DV + ICI combinations (e.g., toripalimab)

Abbreviations: ADC, antibody–drug conjugate; ICI, immune checkpoint inhibitor.

**Table 5:** The clinical activity and survival outcomes of major antibody–drug conjugates (ADCs) with immune checkpoint inhibitors (ICIs) in urothelial carcinoma (UCC).

Therapy	Target	Treatment Setting	ORR	Median PFS	Median OS	Clinical Significance
EV + Pembrolizumab	Nectin-4 + PD-1	1st line/metastatic	68%	12.3 months	23.0 months	First-line standard therapy; high response rate and improved survival
SG + Pembrolizumab	Trop-2 + PD-1	Later-line	41%	5.3 months	12.7 months	Combination strategy; early-stage clinical research
DV + Toripalimab	HER2 + PD-1	1st line/metastatic	76%	13.1 months	31.5 months	First-line option for HER2-positive UC; improved survival outcomes

Abbreviations: ORR, objective response rate; PFS, progression-free survival; OS, overall survival.

## Data Availability

No new data were created or analyzed in this study. Data sharing is not applicable to this article.
